# Utility of Deceased Expanded-Criteria Donors in Kidney Transplantation: A Single-Center Experience †

**DOI:** 10.3390/jcm14093232

**Published:** 2025-05-07

**Authors:** Yavuz Ayar, Alparslan Ersoy, Emel Isiktas Sayilar, Abdülmecit Yildiz, Fatma Ezgi Can, Aysegul Oruc

**Affiliations:** 1 Department of Nephrology, Bursa Faculty of Medicine, Health Sciences University, 16120 Bursa, Turkey; 2 Department of Nephrology, Faculty of Medicine, Bursa Uludag University, 16059 Bursa, Turkey; alpersoy@uludag.edu.tr (A.E.); mecityildiz@gmail.com (A.Y.); aysegul@uludag.edu.tr (A.O.); 3 T.R. Ministry of Health, Ankara Etlik City Hospital, 06170 Ankara, Turkey; emelisiktas@yahoo.com; 4 Department of Biostatistics, Faculty of Medicine, Izmir Katip Celebi University, 35620 İzmir, Turkey; fatmaezgican@gmail.com

**Keywords:** acute rejection, cadaveric transplantation, expanded-criteria donors, mycophenolate mofetil

## Abstract

**Purpose**: The success of solid organ transplantation and the consequent increase in the patients on the waiting list has led to an increased utilization of donor kidneys with a high kidney donor profile index (KDPI)/expanded criteria. In our study, patients who underwent transplantation based on the standard and expanded donor criteria were compared in terms of factors affecting graft survival. Data of patients who underwent transplantation from cadavers with standard and extended criteria (SCD, ECD) between 01 July 2011 and 30 June 2016 were evaluated retrospectively. Donor characteristics, treatment type, response and graft characteristics, 1st-, 3rd-, and 5th-year graft survival, and acute rejection rates were analyzed retrospectively. **Recent findings**: In terms of the causes of death, cerebrovascular accidents were more common in the ECD group (*p* < 0.001). Hypertension and diabetes were more common in both donor groups and were detected more frequently in recipients in the ECD group (*p* < 0.001). The absence of mycophenolate mofetil (MMF) use and the presence of an acute rejection attack adversely affected graft survival at the end of the 1st, 3rd, and 5th years. **Summary**: The utilization of expanded criteria donors is widespread. Appropriate monitoring of patients undergoing immunosuppressive therapy, especially using mycophenolate mofetil (MMF) and the presence of acute rejection, affect graft survival.

## 1. Introduction

The incidence of chronic kidney disease (CKD) has been increasing worldwide with the rise in comorbid diseases (such as hypertension and diabetes), and kidney transplantation remains the priority for patient survival receiving renal replacement therapy (RRT) in CKD. In previous studies, survival after transplantation from a suitable cadaver seems to be superior to other RRTs [1,2,3].

In recent years, the difficulties in finding donors and the decrease in the cadaver pool has increased the waiting time in dialysis and the interest in utilizing expanded-criteria donors. In the USA, before 2001, the rate of cadaveric age ≥60 years was 50%. The rate of cadavers over 60 years of age in France was 26.8% in 2005. In 2002, The Organ Procurement and Transplantation Network (OPTN) defined expanded-criteria donors (ECDs). ECDs were defined as older kidney donors (≥60 years old) or donors who are aged 50–59 years old and have the following two characteristics: hypertension, baseline serum creatinine >1.5 mg/dL, or death from cerebrovascular accidents [2,3,4,5,6,7]. Scoring systems such as the kidney donor profile index (KDPI) and kidney donor risk index (KDRI) are used to assess cadaver quality [8,9].

In our study, we evaluated graft and patient survival and outcomes of cadaveric kidney transplantation according to the ECD and standard-criteria donor (SCD) definitions in our unit, retrospectively.

## 2. Methods

### 2.1. Patients

The data, donor characteristics, recipient treatments, graft characteristics, and clinical and laboratory values of patients who underwent transplantation in our center between 01 July 2011 and 30 June 2016 utilizing ECD (63 patients) and SCD (92 patients) were compared randomly and retrospectively from hospital records. ECDs were described as older kidney donors (> or =60 yr) or donors who are aged 50 to 59 years old and have two of the following three features: hypertension, terminal serum creatinine >1.5 mg/dL, or death from cerebrovascular accident.

The kidney donor risk index (KDRI) score was calculated by evaluating data on age, weight, height, race, creatinine, blood pressure, diabetes, and hepatitis C history. KDRI values were converted to KDPI values using OPTN data [10]. In our study, calcineurin inhibitor (CNI)-based regimens [Kidney Disease: Improving Global Outcomes (KDIGO transplantation guidelines)] were mostly used. As recommended by the guidelines, we attempted to keep the cyclosporine level around 200 ng/mL in the first 3 months, 150–200 ng/mL in the next 6 months, and then 100–150ng/mL until the 2nd year, and 100 ng/mL after the 2nd year. We tried to keep the tacrolimus level in the range of 12–15 ng/mL for the first 3 months, then 10–12 ng/mL for the first 6 months, then 8–10 ng/mL until the 2nd year, and then 5.2–8 ng/mL after the 2nd year. We tried to give as full as possible a dose of mycophenolate mofetil (2000 mg/day for MMF, 1440 mg/day for mycophenolate sodium). The dose was reduced in patients with diarrhea, severe infection, risk of sepsis, thrombocytopenia, severe leukopenia, etc. (according to the Elite-Symphony Trial).

The corticosteroid dose (prednisolone) was reduced to 5 mg/day after 6 months. Switching to mTOR was performed in patients who developed calcineurin toxicity (nephrotoxicity) [11].

Donors who did not meet the condition of being cadavers, such as those with a history of malignancy, high risk of transmission, and active infection, etc., were excluded from the study.

The diagnosis of rejection was made based on clinical findings and kidney biopsy.

CNI toxicity was detected by drug level and kidney biopsy results when kidney function values increased.

Patients who did not have regular follow-ups during the specified period and were transferred to another center were also excluded from the study. We de-identified all patient details [12].

Ethics committee approval was obtained from another center (15–21 January 2019). We allow fellow researchers to reproduce our methodology. Our study was retrospective, and the ethics committee allowed us to use the data in an anonymized form. Since this study was retrospective, patient consent was not obtained following the recommendation of the ethics committee.

### 2.2. Biostatistical Analysis

Data are expressed as means ± SD and median (min–max). Percentage change values were calculated as follows: (postoperative value-preoperative value)/preoperative value for time-dependent values, and these values were used in intergroup comparisons. The Mann–Whitney U test, the independent sample t-test, Pearson’s chi-square test, Fisher’s exact test, and the Fisher–Freeman–Halton test were used for comparisons. Survival times were calculated using Kaplan–Meier analysis, and factors were examined using the log-rank test. Cox’s regression analysis was used to determine the hazard ratios and risk factors. Statistical analyses were performed using the Statistical Package for Social Sciences for Windows [version 22.0; (SPSS, Chicago, IL, USA)]. Statistical significance was set at *p* < 0.05.

## 3. Results

As expected, the mean age of the SCD group was lower than that of the ECD group. The last and 24 h urine volumes were lower in the ECD group. The body mass index (BMI) was higher in the ECD group. The number of cadavers with a history of diabetes and hypertension was higher in the ECD group. Additionally, the cause of death due to trauma was more prominent in the SCD group. The cold ischemia time was longer in patients with ECD. The rates of HT and DM in recipients and the KDPI and KDRI scores were higher in the ECD group. When the recipients were evaluated, the history of HT was higher in transplant recipients from the ECD group. In terms of hospitalization due to infection, acute rejection, and graft and patient survival after transplantation, there was no change in the recipients according to the type of cadaver (Table 1 and Table 2).

The incidence of primary non-functioning kidneys was higher in the ECD group. The first-month creatinine level was higher and the GFR level was lower in the ECD group (Table 3).

No difference was observed between the two groups in terms of the surgical and medical complications observed after transplantation (Table 4 and Table 5).

**Figure 1:**
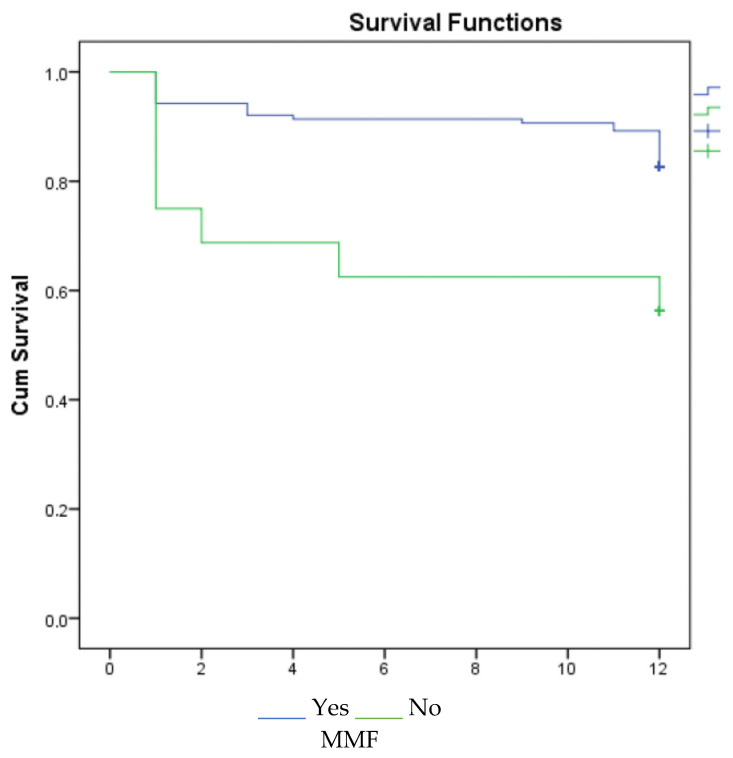

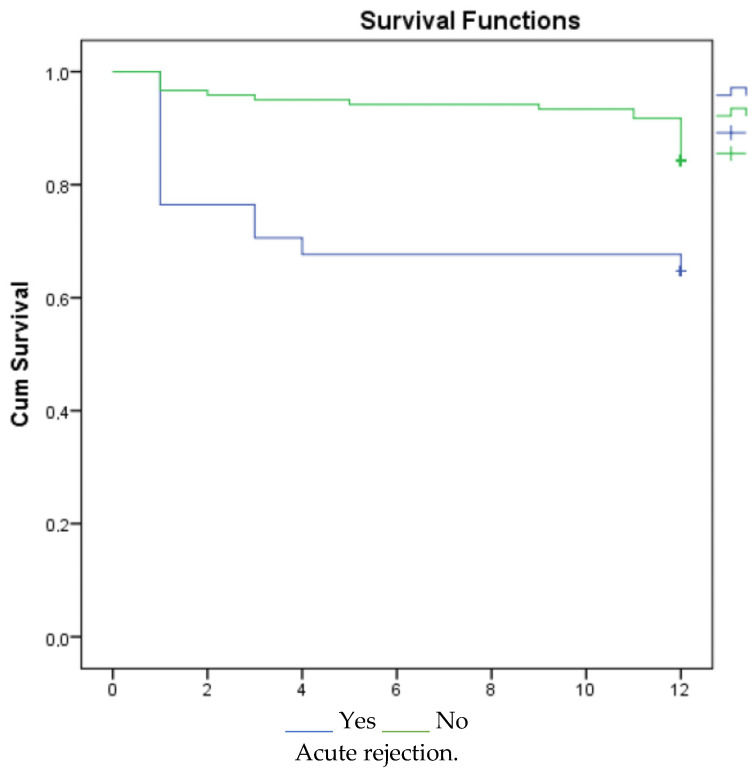
Survival graph for 1 year by MMF and acute rejection.

**Figure 2:**
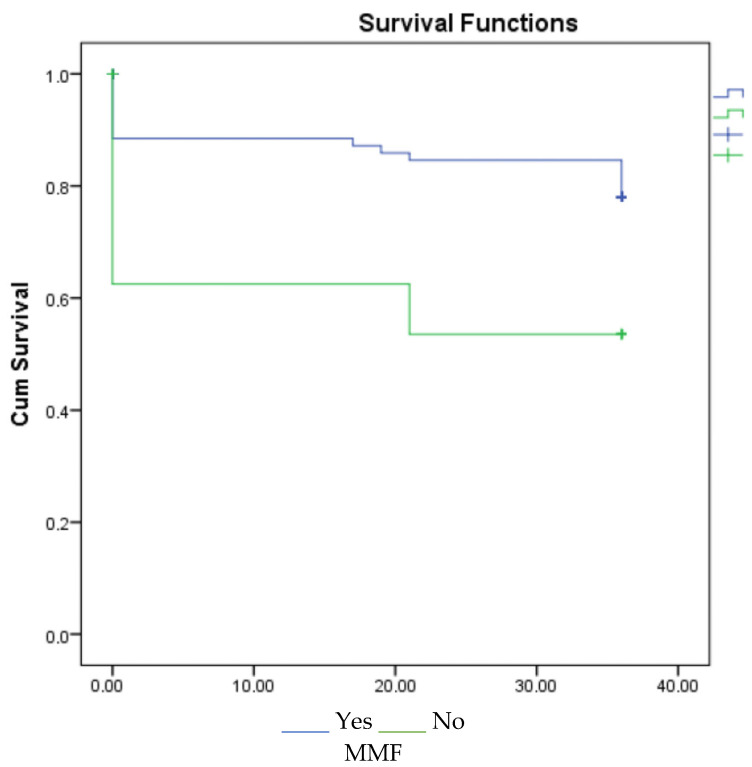

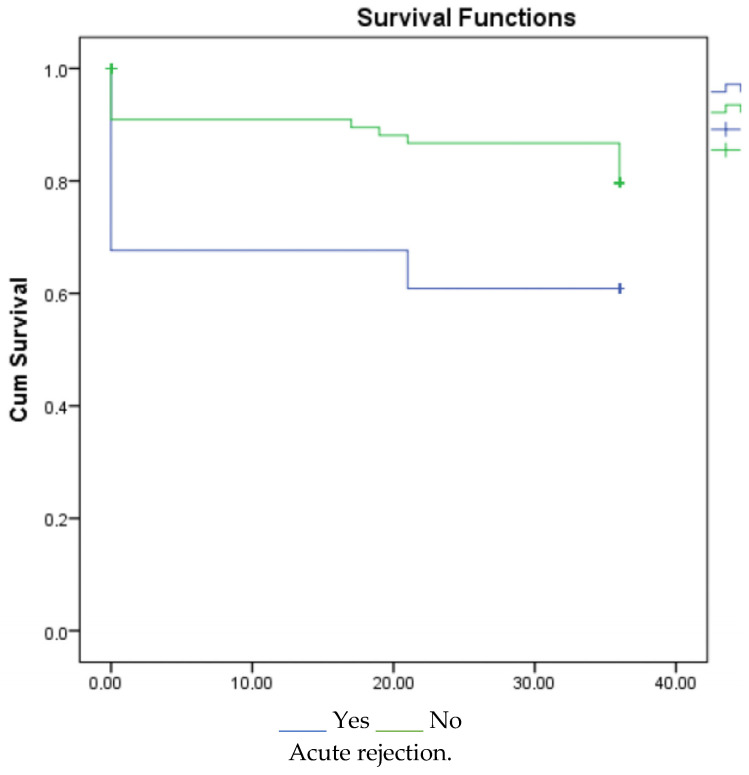
Survival graph for 3 years by MMF and acute rejection.

**Figure 3:**
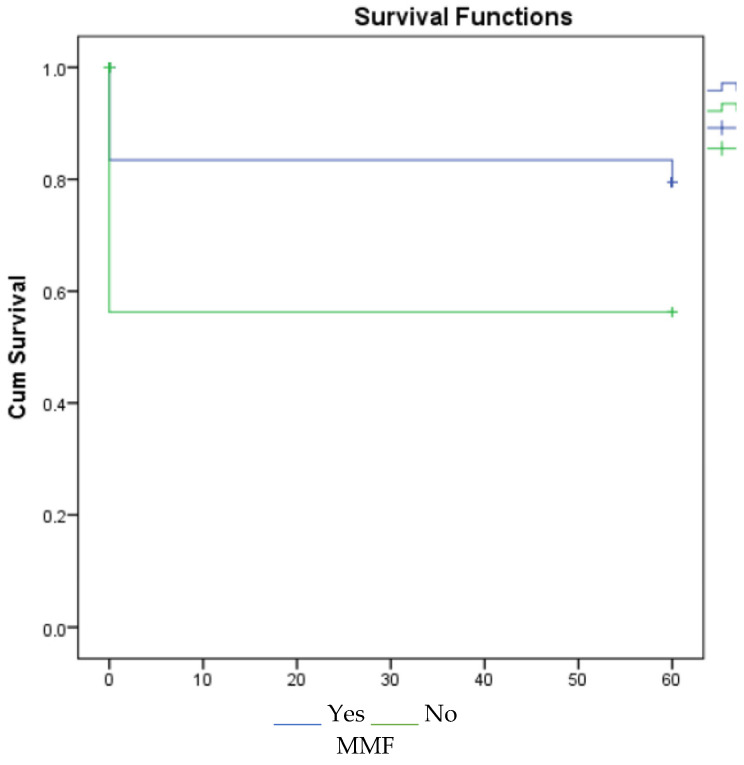

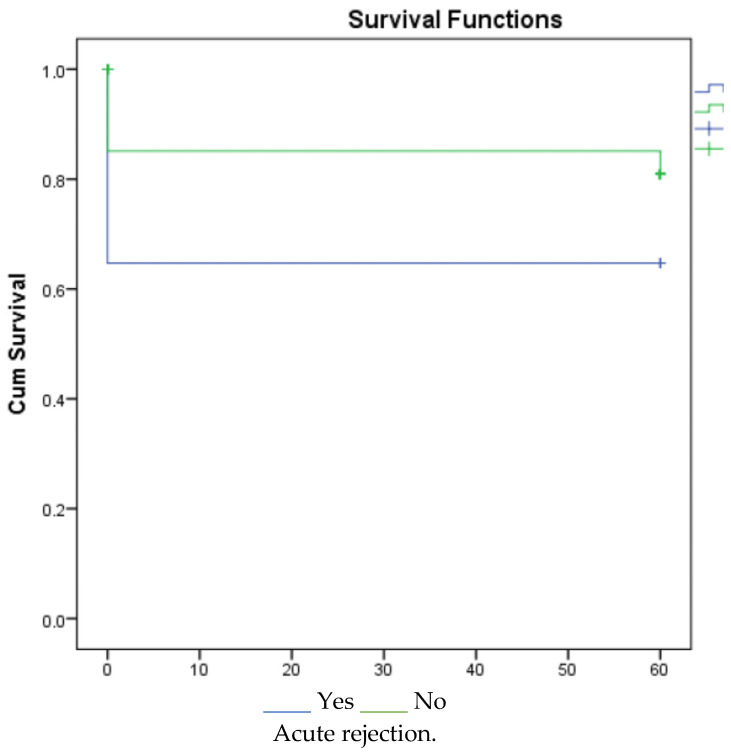
Survival graph for 5 years by MMF and acute rejection.

According to the 1-, 3-, and 5-year survival results (for graft), renal survival was longer in the mycophenolate mofetil (MMF) users, and renal survival was adversely affected in patients with acute rejection (Figure 1, Figure 2 and Figure 3).

When patient survival time was examined for 1-, 3- and 5-year survival for the patients, it was not statistically significant. No significant results were found in the multivariate analysis.

## 4. Discussion

The need for RRT has been increasing due to the increase in the incidence and prevalence of CKD and end-stage renal disease. In kidney transplantation, the need to find donors and cadavers and the length of the waiting time in dialysis have increased. Paired kidney exchange, dual kidney transplantation, and ECD are utilized to meet patient needs.

Considering the global increase in the elderly population, an increase in ECD is expected in the future. In our country’s database, the donor rate between the ages of 45–64 was 36.05%, and it was 5.36% over the age of 65 years. These rates have increased compared to those in the previous year (29.72% and 4.04, respectively) [9,13]. The utility of ECD affects both patient and graft survival [14]. Graft and patient loss were higher in ECD at the end of the 1st and 5th years compared to those transplanted with standard criteria. In a study that evaluated 1554 recipients from Switzerland, patient and graft survival were negatively affected when donor age increased [15,16]. As expected, in our study, age was higher in the ECD group compared to the SCD group.

The incidences of rejection and primary non-functional grafts were higher after the utility of ECD. The incidence of a primary non-functioning kidney was approximately 2% in patients with a KDPI ≥ 80. The rejection rate was 7% by the end of the 6th month. In a study in which 720 cadaveric donors were evaluated, acute rejection rates were higher in patients with a KDPI > 85. In this study, the contribution of the acute rejection rate to graft function at the end of the first year was not significant. In another study, 663 transplants were analyzed, and age-related acute rejections after three months had a negative effect on graft survival at the end of the 1st and 5th years. In addition, rejection rates were similar between the SCD and ECD groups in some studies. Acute rejection rates were high in patients with delayed graft (DGF) function [9,17,18,19,20]. In our study, no change was detected in the rejection rates in both groups. In the regression analysis, the 1st-, 3rd-, and 5th-year graft survivals were low in those who had an acute rejection attack. Rejection is thought to affect graft survival due to the effects of the primary disease and immunosuppressive therapies.

Drug pharmacokinetics in elderly patients should also be considered, particularly with regard to the effectiveness of immunosuppressive therapies. Considering the patient’s clinical and side-effect profiles in terms of immunosuppressive therapies, it was determined that the pharmacokinetics of CNI and steroids changed but MMF did not [21].

However, CNI-free regimens based on reduced CNI exposure and mammalian target of rapamycin inhibitors (mTOR inhs) have shown acceptable results in appropriately selected ECD transplant recipients. CNI-free mTOR inhs-based therapy compared with MMF-based treatment in kidney transplantation with advanced-age donors was associated with an acceptable outcome, but increased proteinuria and acute rejection in mTOR inhs-treated patients was detected more. The best results with mTOR inhs have been found in patients without delayed graft function [22]. In a multicenter study including 3143 patients, the combination of CNI and MMF increased graft survival and decreased acute rejection rates [23]. Studies have shown that MMF plays a key role in immunosuppressive treatment regimens, and rejection and graft survival are adversely affected by dose reduction and discontinuation of the drug [24]. In our study, MMF had a positive effect on 1st-, 3rd-, and 5th-year graft survival.

The most important shortcomings of our study are that it was retrospective, the number of patients was small, and HLA- and donor-specific antibodies could not be tested. In conclusion, acute rejection attacks negatively affect graft survival, and MMF plays an important role in the RRT treatment protocol. Therefore, attention should be given to immunosuppressive treatment and rejection in ECD transplant recipients.

## Figures and Tables

**Table 1:** Comparisons about donor type.

Donor Type	SCD (n = 92)	ECD (n = 63)	*p*
Gender (n, %)			
Male	63 (68.50%)	38 (60.30%)	0.381
Female	29 (31.50%)	25 (39.70%)	
Age (n)	41 (20–59)	62 (42–86)	***p* < 0.001**
Donor height (cm)	170 (150–196)	170 (150–185)	0.133
Donor kg	70 (50–115)	75 (52–115)	0.065
Last urine (cc)	180 (10–1000)	100 (20–1400)	**0.023**
24 h urine (cc)	4255 (750–14,950)	3200 (800–7500)	**0.030**
Donor BMI	24.71 ± 3.24	26.96 ± 3.64	***p* < 0.001**
DM in donor (n, %)			
Yes	0 (0%)	27 (42.90%)	***p* < 0.001**
No	92 (100%)	36 (57.10%)	
HT in donor (n, %)			
Yes	8 (8.70%)	59 (93.70%)	***p* < 0.001**
No	84 (91.30%)	4 (6.30%)	
Cause of death (n, %)			
CVA	62 (67.40%)	60 (95.20%)	***p* < 0.001**
Trauma	24 (26.10%)	1 (1.60%)	
Other	6 (6.50%)	2 (3.20%)	
İntensive care Hospitalization (day)	3 (1–21)	3 (1–10)	0.134
Cold ischemia (hour)	10 (1–21)	12 (4–36)	**0.020**
HbsAg (n, %)			
Positive	2 (2.20%)	3 (4.80%)	0.397
Negative	90 (97.80%)	60 (95.20%)	
antiHbs (n, %)			
Positive	39 (42.40%)	53 (57.60%)	0.241
Negative	20 (31.70%)	43 (68.30%)	
Yes	8 (8.70%)	59 (93.70%)	***p* < 0.001**
No	84 (91.30%)	4 (6.30%)	
KDPI (%)	56 (19–88)	97 (65–100)	**<0.001**
KDRI (%)	1.06 (0.73–1.57)	1.93 (1.16–3.50)	**<0.001**
HLA mismatch (n)	2 (1–6)	2 (1–6)	0.865

SCD: Standard criteria of donor. ECD: Expanded criteria of donor. DM: Diabetes mellitus. BMI: Body mass index. CVA: Cerebrovascular accident. HT: Hypertension. HbsAg: Hepatitis B surface antigen. antiHbs: Hepatitis B surface antibody. KDPI: Kidney donor profile index. KDRI: Kidney donor resistive index. CMV: Cytomegalovirus.

**Table 2:** Recipient profile/demographics.

Recipient Age (n)	SCD (n = 92) 42.50 ± 13.27	ECD (n = 63) 42.84 ± 14.34	0.879
HT in recipient (n, %)			
Yes	0 (0%)	27 (42.90%)	***p* < 0.001**
No	92 (100%)	36 (57.10%)	
DM in recipient (n, %)			
Yes	19 (20.70%)	16 (25.40%)	0.618
No	73 (79.30%)	47 (74.60%)	
Hospitalization after Tx (n, %)			
UTI	20 (21.70%)	16 (25.40%)	0.926
Pneumonia	25 (27.20%)	15 (23.80%)	
Surgical complication	11 (12%)	9 (14.30%)	
Rejection	8 (8.70%)	6 (9.50%)	
Other	8 (8.70%)	3 (4.80%)	
None	20 (21.70%)	14 (22.20%)	
Graft loss (n, %)			
Yes	12 (13%)	10 (16.10%)	0.763
No	80 (87%)	52 (83.90%)	
Survey			
Alive	76 (82.60%)	48 (76.20%)	0.162
Rejection	5 (5.40%)	9 (14.30%)	
Death	11 (12%)	6 (9.50%)	

Tx: Transplantation. UTI: Urinary tract infection.

**Table 3:** Comparisons about primer treatments.

	SCD (n = 92)	ECD (n = 63)	*p*
CNI (n, %)			
Cyclosporine	49 (53.30%)	26 (41.30%)	0.142
Tacrolimus	43 (46.70%)	37 (58.70%)	
MMF (n, %)			
Yes	84 (91.30%)	55 (87.30%)	0.592
No	8 (8.70%)	8 (12.70%)	
mTOR (n, %)			
Yes	6 (6.50%)	6 (9.50%)	0.549
No	86 (93.50)	57 (90.50%)	
Primary non-functioning kidney (n, %)			
Yes	0 (0%)	5 (7.90%)	**0.010**
No	92 (100%)	58 (92.10%)	
Acute rejection (n, %)			
Yes	19 (20.70%)	15 (23.80%)	0.788
No	73 (79.30%)	48 (76.20%)	
DGF (n, %)			
Yes	13 (14.10%)	14 (22.20%)	0.276
No	79 (85.90%)	49 (77.80%)	
ATN (n, %)			
Yes	31 (33.70%)	20 (31.70%)	0.936
No	61 (66.30%)	43 (68.30%)	

CNI: Calcineurin inhibitors. MMF: Mycophenolate mofetil. mTOR: The mammalian target of rapamycin. DGF: Delayed graft function. ATN: Acute tubular necrosis.

**Table 4:** Comparisons about serum creatinine and GFR.

	SCD (n = 92)	ECD (n = 63)	*p*
Serum creatinine first month (mg/dL)	1.30 (0.53–8.90)	1.61 (0.46–14.80)	**0.014**
Serum creatinine sixth month *	0.08 (−1:1.28)	−0.03 (−0.84:1.15)	0.061
Serum creatinine twelfth month *	−0.02 (−1:3.24)	0.01 (−0.85:5.96)	0.208
Serum creatinine final*	−0.04 (−1:9.34)	0 (−0.82:6.01)	0.510
GFR first month (ml/min/m^2^)	57.67 (7–202)	44 (3.57–202)	**0.005**
GFR sixth month *	−0.07 (−1:65.26)	0.04 (−0.59:4.13)	**0.010**
GFR twelfth month *	−0.05 (−1:7.18)	−0.07 (−0.90:5.66)	0.572
GFR final month *	−0.07 (−1:6.73)	−0 (−0.90:4.77)	0.445

* Percentage change values were calculated and compared. GFR: Glomerular filtration rate.

**Table 5:** Post-transplant surgical and medical complications.

	SCD (n = 92)	ECD (n = 63)
Serum urea (mg/dL)	42 (6–114)	57 (15–137)
Serum creatinine (mg/dL)	1.10 (0.47–6.20)	1.30 (0.50–4.02)
Hgb (g/dL)	11.10 (6.50–17.40)	12.60 (7.42–15.50)
Wbc (10^3^/µL)	14.19 (1.10–29.20)	14 (1.51–46)
Na (mmol/L)	151.50 (12–199)	153 (68–211)
K (mmol/L)	3.69 (1.50–6.40)	3.60 (2.40–4.90)
Post-transplant surgical complications		
Lymphocele	31 (33.70%)	21 (33.30%)
Hernia	1 (1.10%)	3 (4.80%)
İleus	2 (2.20%)	0 (0%)
Other	11 (12%)	8 (12.70%)
None	47 (51.10%)	31 (49.20%)
Post-transplant medical complications		
Drug	3 (3.30%)	3 (4.80%)
Hyperparathyroidism (secondary or tertiary)	2 (2.20%)	1 (1.60%)
TXGMP	1 (1.10%)	0 (0%)
AVN	9 (9.80%)	2 (3.20%)
None	77 (83.70%)	57 (90.50%)

Hgb: Hemoglobin. Wbc: White blood cells. Na: Sodium. K: Potassium. TXGMP: Transplant glomerulopathy. AVN: Avascular necrosis.

## Data Availability

The raw data supporting the conclusions of this article will be made available by the authors on request.
